# Surgical treatment of valvular and supravalvular aortic stenosis in homozygous familial hypercholesterolemia

**DOI:** 10.1007/s11748-014-0378-x

**Published:** 2014-02-08

**Authors:** Hisashi Sato, Masaru Yoshikai, Kazuyuki Ikeda, Yosuke Mukae

**Affiliations:** Department of Cardiovascular Surgery, Shin-Koga Hospital, Tenjin-machi 120, Kurume, Fukuoka 830-8577 Japan

**Keywords:** Homozygous familial hypercholesterolemia, Aortic valve stenosis, Supravalvular aortic stenosis

## Abstract

A 61-year-old male with homozygous familial hypercholesterolemia presented with dyspnea and syncope. He had been treated with low-density lipoprotein apheresis for 26 years. Echocardiography and computed tomography showed severe valvular and supravalvular aortic stenosis. Computed tomography and cardiac catheterization revealed a severely calcified narrowed aortic root and an occlusion in the proximal right coronary artery. During surgery, the ascending aorta was replaced under deep hypothermic circulatory arrest without aortic cross-clamping. After that, the aortic root from the annulus to the sino-tubular junction was enlarged with a two-ply bovine pericardial patch. An aortic valve replacement with a 17 mm mechanical valve and coronary artery bypass grafting to the right coronary artery were performed. The patient recovered from the surgery without any cerebrovascular complications.

## Introduction

Homozygous familial hypercholesterolemia (HFH) is an extremely rare disease, estimated to affect one patient per million in the general population [1]. HFH is mainly caused by a low-density lipoprotein (LDL) receptor gene mutation. A high level of plasma LDL causes significant premature cardiovascular disease, including atherosclerotic coronary artery disease (CAD), and valvular and supravalvular aortic stenosis (AS). We herein report a case with HFH who presented with severe aortic root stenosis and CAD.

## Case report

**Fig. 1:**
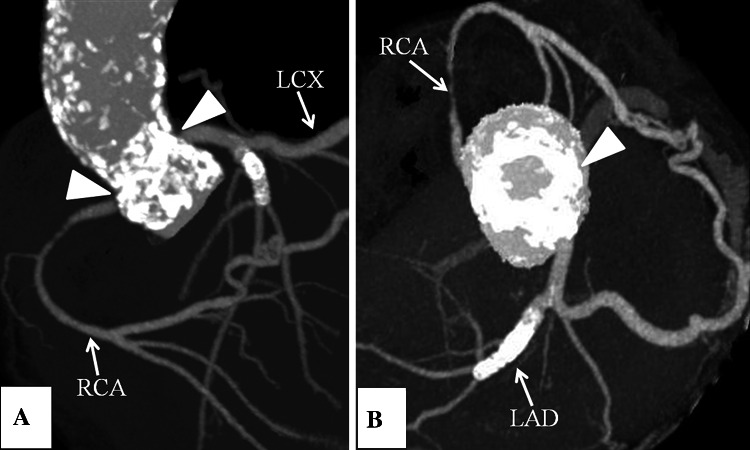
Computed tomography images showing severely calcified aortic root. A calcified plate at the STJ (*arrow heads*) made supravalvular aortic stenosis. **a** Long axis view, **b** Short axis view. *LAD* left anterior descending coronary artery, *LCX* left circumflex coronary artery, *RCA* right coronary artery

A 61-year-old male with HFH had been treated with LDL apheresis for 26 years. He presented with syncope and chronic congestive heart failure. Echocardiography revealed the progression of AS with a peak pressure gradient (PG) of 125 mmHg, mean PG of 76 mmHg, an aortic valve area of 0.36 cm^2^ and moderate aortic regurgitation. The diameters of the aortic annulus, sinus of Valsalva, and sino-tubular junction (STJ) were 17, 25, and 17 mm, respectively. Computed tomography showed the severe calcification of the whole aortic root with protrusion of the calcified plate at the STJ (Fig. 1). Coronary angiography showed an occlusion in the right coronary artery.

**Fig. 2:**
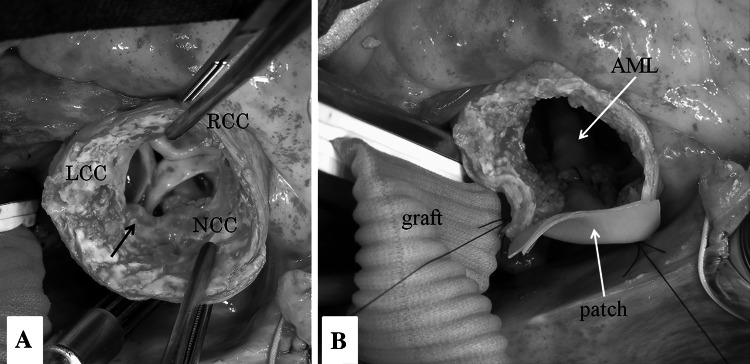
Operative findings. **a** A calcified plate protruded at the STJ, which narrowed the aorta to 17 mm at the STJ level (*arrow*). The aortic valve was severely calcified. **b** Aortic root was enlarged by sewing a bovine pericardial patch from the annulus to the STJ. *RCC* right coronary cusp, *LCC* left coronary cusp, *NCC* non-coronary cusp. *AML* anterior leaflet of the mitral valve

**Fig. 3:**
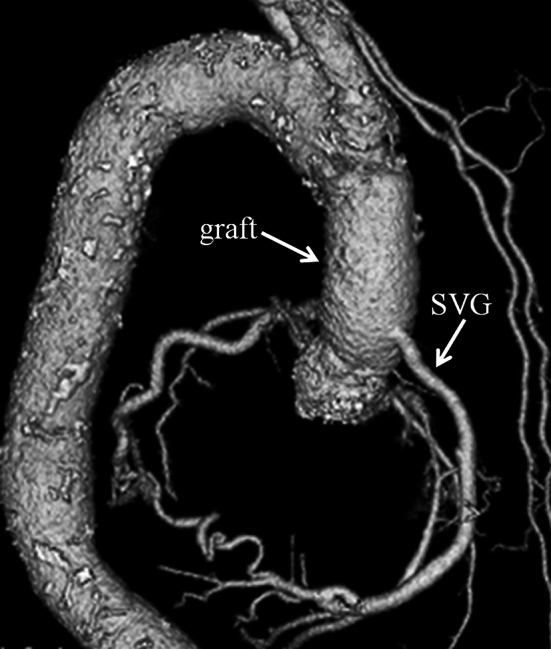
Post-operative 3D-CT image on the posterior view demonstrating well-enlarged aortic root, patent saphenous vein graft (SVG) and the ascending aorta replaced with a graft

During surgery, epiaortic echography demonstrated a heavily calcified aorta and a mobile plaque on the posterior wall of the ascending aorta, so extracorporeal circulation (ECC) was established between a graft anastomosed to the right axillary artery and the bicaval cannulae. At first, the great saphenous vein was anastomosed to the right coronary artery with the heart beating. Under deep hypothermic circulatory arrest (DHCA), retrograde cerebral perfusion was initiated via the superior vena cava. The ascending aorta was incised, and cardioplegic solution was delivered to the left coronary artery and the great saphenous vein to the right coronary artery. The aorta was transected just proximal to the brachiocephalic artery without aortic cross-clamping. A 24 mm one-branched vascular graft was anastomosed to the aortic stump, and ECC was restarted via a branch of the graft. The calcified plate at the STJ protruded so as to narrow the aortic lumen to 17 mm (Fig. 2a). Decalcification of the STJ was carried out using a Cavitron Ultrasonic Surgical Aspirator (CUSA). The aortic valve was severely calcified, as was the tricuspid. The cusps were removed, and the annular calcification was removed by a CUSA. The aortic annulus was too narrow to implant an adequate-sized prosthesis, so the annulus was incised on the intervalvular fibrous trigone. The aortic root was enlarged by sewing a two-ply bovine pericardial patch from the incised annulus to the STJ (Fig. 2b). A St. Jude Medical regent valve (17 mm; St. Jude Medical, Inc., St. Paul, Minn) was implanted on the enlarged annulus. The graft was anastomosed to the proximal aorta just above the STJ. Finally, the great saphenous vein was anastomosed onto the graft. The patient was then successfully weaned from ECC. The period of DHCA, ECC, aortic cross-clamp, operation was 31, 282, 179 and 515 min, respectively. The lowest rectal temperature was 20.5 °C. He recovered favorably after the operation without any cerebrovascular complications. Post-operative echocardiography revealed that a peak PG from the left ventricle to the aorta was 19 mmHg, a mean PG was 7 mmHg and an aortic valve area was 1.37 cm^2^. Post-operative 3D-CT demonstrated well-enlarged aortic root and patent saphenous vein graft (Fig. 3). One year later after surgery, he is in New York Heart Association functional class I.

## Discussion

We herein presented a surgical case of HFH, who had CAD, valvular and supravalvular AS, and an atherosclerotic ascending aorta. HFH causes premature cardiovascular diseases including CAD, valvular and supravalvular AS, and atherosclerosis and/or calcification of the arteries, including the ascending aorta, which makes surgical treatment difficult.

A calcified aorta and atheromatous ascending aorta are associated with a risk of developing cerebrovascular complications during cardiovascular surgery which requires ECC. Yasuda et al. [2] previously performed an endarterectomy of the ascending aorta under DHCA without aortic cross-clamping, and then replaced a stenotic aortic valve during aortic cross-clamping for a patient with HFH. We used the right axillary artery for the arterial inflow of ECC, and replaced the ascending aorta without aortic cross-clamping during DHCA with retrograde cerebral perfusion, because severe calcification and a mobile plaque were present in the ascending aortic wall. Cannulation and cross-clamping of a calcified atheromatous aorta should be avoided to prevent cerebrovascular complications. Moreover, we think that the ascending aorta should be replaced without aortic cross-clamping, when there is mobile plaque on the ascending aorta.

Not only valvular AS, but also supravalvular AS and a narrowed aortic annulus are problems for patients with HFH. When the annulus is of sufficient size for adequate prosthetic valve implantation, a resection of the protruding calcification at the STJ and an aortic valve replacement were the treatment of choice, as described by Yasuda et al. [2]. In addition to the presence of supravalvular stenosis, when the annulus is also narrow, a valve replacement with an annular enlargement procedure or a full root replacement is an alternative. Saito et al. [3] replaced an aortic valve after an annular enlargement procedure because of a severely calcified aortic wall around the coronary ostia. In the present case, the wall of the sinus Valsalva was severely calcified, so we abandoned the full root replacement, and enlarged the aortic annulus by incising the intervalvular fibrous trigone. The sinus Valsalva and STJ could also be enlarged with the patch that had been used to enlarge the annulus during the valve replacement.

The surgical treatment for valvular diseases in HFH patients is very complex, and necessitates a prolonged myocardial ischemic time. Moreover, almost all patients with HFH who undergo cardiac surgery have CAD. Selective delivery of cardioplegic solutions to each coronary artery may be difficult because of the calcified aortic wall around the coronary artery ostia. In these circumstances, anastomosing a free graft to the stenotic coronary artery before aortic cross-clamping and/or retrograde cardioplegia via the coronary sinus is advisable for myocardial protection.

## Conclusion

The surgical treatment for HFH is associated with many problems, and is technically demanding. To accomplish such a high-risk operation safely, special attention should be paid to the establishment of ECC, manipulating the ascending aorta, procedures on the narrowed aortic root, and to myocardial protection.
